# Anti‐NMDA Receptor Encephalitis in a Young Adult with Neuropsychiatric and Cognitive Symptoms‐ A Case Study

**DOI:** 10.1002/alz70857_102505

**Published:** 2025-12-24

**Authors:** Aishwarya Jaikrishnan, Nirumal Khumar, Nooka Monika Reddy, Sathish Kumar Mani, Praveen Nandha Kumar Pitchan Velammal

**Affiliations:** ^1^ Saveetha Medical College and Hospital, Chennai, TamilNadu, India; ^2^ Saveetha Medical College and Hospital, Chennai, Chennai, TamilNadu, India; ^3^ Tirunelveli Medical College, Tirunelveli, Tirunelveli, TamilNadu, India

## Abstract

**Background:**

Anti‐NMDA receptor encephalitis is an autoimmune disorder that affects the brain, leading to a combination of neuropsychiatric symptoms, including severe mood changes, agitation, and psychosis, as well as cognitive impairments resembling dementia. It is commonly seen in young individuals and requires early intervention to prevent long‐term neuropsychiatric deficits.

**Method:**

Case Study

**Result:**

A 20‐year‐old male, born from a non‐consanguineous marriage, presented with a history of multiple episodes of new‐onset focal seizures, which were followed by well‐formed visual hallucinations. Over a period of days, he developed significant behavioral changes, including agitation, anger outbursts, memory disturbances, and a marked decrease in interest in daily activities. His speech output also diminished progressively and eventually led to a mute state. The patient showed increased slowness in activities and a general decline in functional ability. On neurological examination, the patient was found to be in a severe catatonic state, unable to communicate or engage in any purposeful movements. Perioral dyskinesias, characterized by involuntary movements of the mouth, were noted. The remainder of the examination, including cranial nerve function, motor, sensory, cerebellar, and extrapyramidal assessments, was grossly normal. Further investigations included a plain MRI of the brain, which showed a left temporal hyperintensity, suggestive of inflammation, though no other structural abnormalities were identified. EEG was normal, which is not uncommon in early stages of this condition. CSF analysis was normal, with no signs of infection or inflammatory changes. An autoimmune panel was sent, and NMDA receptor antibodies were found to be positive, confirming the diagnosis of anti‐NMDA receptor encephalitis. The patient was started on intravenous immunoglobulin (IVIG) therapy, resulting in a significant improvement in his clinical condition, including resolution of catatonia and gradual restoration of speech and cognitive function.

**Conclusion:**

Early recognition of anti‐NMDA receptor encephalitis, characterized by neuropsychiatric and cognitive symptoms, is critical. Timely treatment with immunotherapy like IVIG can significantly improve recovery and prevent irreversible cognitive decline, highlighting the importance of prompt diagnosis in managing this potentially reversible form of encephalitis.

